# Saturated Fatty Acids Modulate Autophagy’s Proteins in the Hypothalamus

**DOI:** 10.1371/journal.pone.0119850

**Published:** 2015-03-18

**Authors:** Mariana Portovedo, Letícia M. Ignacio-Souza, Bruna Bombassaro, Andressa Coope, Andressa Reginato, Daniela S. Razolli, Márcio A. Torsoni, Adriana S. Torsoni, Raquel F. Leal, Licio A. Velloso, Marciane Milanski

**Affiliations:** 1 Faculty of Applied Science, University of Campinas, UNICAMP, Limeira, Brazil; 2 Obesity and Comorbidities Research Center, University of Campinas, UNICAMP, Campinas, Brazil; 3 Faculty of Nutrition, Federal University of Mato Grosso, UFMT, Cuiaba, Brazil; State University of Rio de Janeiro, Biomedical Center, Institute of Biology, BRAZIL

## Abstract

Autophagy is an important process that regulates cellular homeostasis by degrading dysfunctional proteins, organelles and lipids. In this study, the hypothesis that obesity could lead to impairment in hypothalamic autophagy in mice was evaluated by examining the hypothalamic distribution and content of autophagic proteins in animal with obesity induced by 8 or 16 weeks high fat diet to induce obesity and in response to intracerebroventricular injections of palmitic acid. The results showed that chronic exposure to a high fat diet leads to an increased expression of inflammatory markers and downregulation of autophagic proteins. In obese mice, autophagic induction leads to the downregulation of proteins, such as JNK and Bax, which are involved in the stress pathways. In neuron cell- line, palmitate has a direct effect on autophagy even without inflammatory activity. Understanding the cellular and molecular bases of overnutrition is essential for identifying new diagnostic and therapeutic targets for obesity.

## Introduction

Obesity is the result of a disproportionately high energy intake compared to energy expenditure and is triggered by a complex interaction between the environmental and genetic factors [1–3]. Excessive calories in dietary consumption, particularly of long-chain saturated fatty acids, can result in the development of metabolic dysfunction and increased body weight [4].

The hypothalamus is the main area of the brain that controls energy intake and expenditure [5, 6]. In animals, chronic intake of a high-fat diet (HFD) causes diet-induced obesity (DIO), which results in leptin and insulin resistance in hypothalamic neurons [3, 5, 6]. Increased inflammation in the hypothalamus was identified to mediate the development of obesity and the pathways include IKKB/NF-kB pathway [7, 8] and upstream inputs such as MyD88 [4, 9], endoplasmic reticulum stress [4, 7, 8], and JNK signaling [10–13]. Chronic inflammatory stimuli can also lead to neuronal apoptosis which is important for the anorexigenic response [14–15]. Recently, neuroimaging studies revealed that dysfunction and neuronal loss were associated with obesity in the hypothalamus of humans and rodents [16–18].

In addition to the effects on food intake and energy expenditure, hypothalamic inflammation seems to impair systemic glucose metabolism. Genetic and pharmacological modulation of the endoplasmic reticulum stress and inflammation pathways in the hypothalamus, affected liver gluconeogenesis [8, 19–20]. Inflammatory inhibition of TLR4 or TNFα signaling in the hypothalamus improved insulin signal transduction in the liver and reduced hepatic glucose production [4]. These studies suggest that hypothalamic inflammation plays a role in weight gain and systemic dysfunction of glycemic control. Meng and Cai have demonstrated that neuronal autophagy is compromised under conditions of chronic excess fatty acids in the diet. In chow-feeding mice, the site-specific inhibition of ATG7 in the mediobasal hypothalamus leads to autophagy inhibition, impairment of hypothalamic control of energy balance, obesity and hypothalamic inflammation via IkB activation. These metabolic changes were exacerbated under HFD feeding with progression of insulin and leptin resistance [21]. Here the hypothesis that malfunction of the macroautophagy (hereafter referred to as autophagy) system contributes to the impairment of the hypothalamic regulation of the body’s energy balance in a time-dependent manner was explored. Autophagy is a homeostatic process in every eukaryotic cell and it is responsible for the degradation of damaged proteins and organelles. Autophagy also sequesters the cytoplasmic components in the double-membrane vesicles called autophagosomes [22]. The autophagosomes subsequently fuse with lysosomes, where the damaged proteins and organelles are degraded by lysosomal proteases and recycled [22–24]. Impaired autophagy may lead to inflammation which suggests that autophagy contributes to the inhibition of the inflammatory response [21, 25–26].

In this study, mice were chronically exposed to a high-fat diet which increased the expression of inflammatory markers and resulted in a loss of basal autophagy. Rapamycin injection intracerebroventricular (icv,) activated autophagy in the hypothalamus and improved glucose tolerance in obese animals without altering body weight. This intervention decreased markers of cellular stress in the hypothalamus of obese animals while icv treatment with palmitic acid contributed to decreased autophagy efficiency in this brain region. Investigating the cellular and molecular basis of autophagy is crucial in identifying new diagnostic and therapeutic targets for obesity.

## Materials and Methods

### Antibodies, chemicals and primers

Antibodies against LC3B (ab48394, AbCam, Cambridge, MA, USA), p62 (ab91526), pEIF2S1 (ab32157), pJNK (sc-6254, Santa Cruz Biotechnology, Santa Cruz, CA), EIF2 (ab32157), Bax (sc493), Bcl-2 (sc492), Anti rabbit IgG (GE 30021019), Goat-anti-Mouse IgG (Zymed 626520), Rabbit-anti-Goat IgG (Zymed 811620) and Beta actin (ab8227) were used for western blotting. For immunostaining, Vectashield Mounting Medium with DAPI (Vector laboratories) and LC3B (Cell signaling 3868, Boston, MA, USA) were used. Primers for mouse GAPDH, TNFR1, IL10, IL1β, MAP1LC3, ATG5, SQSTM1, BECN-1 and ULK1 (Applied Biosystems) were used for reverse-transcriptase polymerase chain reaction (RT-PCR). Autophagy was induced with Rapamycin (Cayman Chemical Company).

### Experimental animals

Seven-week-old male Swiss mice were provided by the University of Campinas Animal Breeding Center (CEMIB, Brazil). All animal procedures followed the *Guide for the Care and Use of Laboratory Animals* published by National Institute of Health [27] and the guidelines of the Brazilian College for Animal Experimentation. The experiment was approved in advance by the State University of Campinas Ethics Committee (Protocol 2623–1). Animals were maintained on a 12 hour light-dark cycle and each mouse was individually housed in cages (22°± 1°C) polypropylene microisolators with *ad libitum* food and water. Mice were randomly divided and received a chow diet or a high-fat diet that in which the main fat source is lard for 8 or 16 weeks. The control diet has the following macronutrient composition: ∼ 70, 20 and 4 g/kg of carbohydrate, protein and lipid, respectively, and the HFD, ∼ 35, 20 and 35.2 g/kg of carbohydrate, protein and lipid, respectively. The effects of rapamycin were evaluated in mice following stereotaxic surgery to implant a cannula, for icv injections. The cannula was made with hypodermic needle size 27G. Food ingestion and body mass were monitored weekly with a balance (Bel Engineering, Monza, Italy) in mice that did not have surgery while icv treated mice had their food ingestion and body weight assessed daily.

### Cannula implantation and icv treatment

Mice receiving chow diet underwent stereotaxic surgery to implant a cannula into the lateral ventricle (from Bregma: anterior-posterior, 0.34 mm; lateral 1.0 mm; and depth: 2.2 mm) according to a previously described method [28]. For this, mice were anesthetized (i.p Cetamin 0.1 g/kg; Diazepan 5mg/kg; Xilasin 3 mg/kg), and during the 7 days of recovery they received sodium dipyrone analgesia. The cannula placement was tested by measuring the dipsogenic response to an icv injection of angiotensin II (2 μL of a 10^−6^ M solution) (Sigma, St. Louis, MO, USA). Mice which received previously high fat diet for 16 weeks were treated with Rapamycin, an autophagy inductor. The drug was dissolved in DMSO to obtain a 5 mM concentration and in saline to achieve a final concentration of 25 μM. Animals were treated once a day with the rapamycin solution (2 μL) for 6 days. Body weight and food intake were monitored daily.

### Protein extraction and immunoblotting

Mice were anesthetized (i.p Cetamin 0.1 g/kg; Diazepan 5mg/kg; Xilasin 3 mg/kg), decapitated and the hypothalamus was excised. The tissues were homogenized in a Triton X-100 buffer with an anti-protease cocktail using a tissue homogenizer (Polytron-Aggregate, Kinematica, Littau/Luzern, Switzerland). Protein concentration was determined with a dye-binding protein assay kit (Bio-Rad Laboratories, Hercules, CA).

Immunoblotting was performed by using protein extracti (30 μg) from each mouse sample which was incubated for 5 minutes at 95°C with Laemmli buffer (1 mmol sodium phosphate/L, pH 7.8, 0.1% bromophenol blue, 50% glycerol, 10% Sodium Dodecyl Sulfate (SDS), 2% mercaptoethanol). Polyacrylamide gels (SDS-PAGE) were used to separate the proteins in each sample. Then a Trans Blot SD Semi-Dry Transfer Cell (Bio-Rad) was used to transfer the samples from the gel to a nitrocellulose membrane (Bio-Rad) by semi-dry blotting (20V for 30 min) the gel in a transfer buffer that contained methanol and SDS. Membranes were then blocked with a solution containing 5% skim milk in Tris-buffered saline (TBS)-Tween 20 (TTBS; 10 mmol Tris/L, 150 mmol NaCl/L, 0.5% Tween 20) for 2 h at room temperature. The membranes were incubated overnight with primary antibodies at 4°C, which was followed with a 1-hour incubation of horseradish peroxidase-conjugated secondary antibody at room temperature. Bands were detected by chemiluminescence (Thermo Scientific #34078) and quantified by densitometry (UN-Scan-it Gel 6.1, Silk Scientific Inc, Orem, Utah USA).

### Immunofluorescence staining

The brain was excised after the mice were decapitated. Each SNC was fixed in 4% paraformaldehyde and each hypothalamus was processed for paraffin embedding and sectioned into 5.0-μm sections. Samples were incubated with primary antibodies overnight and with secondary antibodies conjugated to FITC or rhodamine for 2 hours (sc2777and sc2092, respectively; Santa Cruz Biotechnology, Santa Cruz, CA). The DAPI stain was used for nuclear staining while the Leica FW 4500 B microscope captured the images. Hypothalamic areas were observed according to the landmarks in the mouse brain atlas [29]. Analysis and documentation of the results were performed using Leica Application Suite V3.6 (Switzerland).

### Glucose tolerance test

Blood samples collected from the tail vein were used to determine the blood glucose levels (time 0). Glucose tolerance tests were performed on the mice after a 4-hour fast. The animals were then administered an intraperitoneal injection of glucose 1 g glucose/ kg body weight and the blood glucose levels were measured after 15, 30, 60, 90, 120 and 150 minutes.

### RNA extraction and qRT-PCR

Hypothalamic tissue was homogenized in Trizol (Invitrogen 15596018, Sao Paulo, Brazil), the total RNA, including mRNA, was isolated and quantified (Nanodrop 8000, Thermo Scientific, Wilmington, DE, USA). The cDNA was synthesized from the mRNA by using 3 μg of the total RNA sample with a High-Capacity cDNA Reverse Transcription Kit (Applied Biosystems 4368813). QRT-PCR analysis was performed in an ABI Prism 7700 sequence detection system (Applied Biosystems), which used a GAPDH primer as an endogenous control. Each PCR contained 3.0 ng of the reverse-transcribed RNA, 200 nM of each primer, TaqManTM (Applied Biosystems) and RNase free water. Data was analyzed with the 7500 System SDS Software (Applied Biosystems, Life Technologies).

### Cell culture

CLU-189 adult mouse hypothalamic cell line and BV-2 mouse microglia cell line were maintained in DMEM with 4.5 mg/L of glucose, containing 10% fetal bovine serum (FBS), 100 u/mL penicillin and 100 mg/mL streptomycin. In all experiments, cells were incubated with DMEM 4.5 mg/L containing 1% FBS and plated at a final concentration of 85% cells/plate. BV-2 cell line was divided into control group (167 μM of BSA), or PALM (500 μM of palmitic acid and 167 μM BSA incubated for 2 h at 37°C to solubilize prior the incubation in BV2 cells) for 6, 12 or 24 hours. The conditioned media of BV2 cells were collected and used to incubate the CLU-189 cells. In some experiments, we wanted to evaluate the isolated effect of palmitic acid in hypothalamic neurons. For that, we incubated CLU-189 cell line with palmitic acid (500 μM of palmitic acid and 167 μM of BSA, homogenized for two hours at 37°C prior the incubation) or the BSA control (167 μM of BSA) for 6, 12 or 24 hours. The expression/activation of proteins involved in inflammation was evaluated by Real-Time PCR or western Blot.

### Statistical Analysis

In order to determine the sample size, pre-experimental evaluation was conducted using stable and unstable variables. Variables that had the greatest deviations were repeated in order to provide the credibility of the average value obtained. Unstable variables do not require a large number of repetitions. Moreover, we used a reduced sample number for rapamycin icv injection experiments, due to the time of treatment with diet and the need for surgery. For all experiments, the animals were distributed in a completely randomized block design in order to obtain reliable and stable data. Four to five animals from at least two or three different litters were used. Results are expressed as mean values +/- SEM. Levene’s test for the homogeneity of variance determined if the data could be used for parametric analysis of variance. Student’s unpaired t-tests were used to compare the differences between two groups. When there were more than two groups, a One-Way ANOVA was performed. Two-Way ANOVAs were applied when necessary. Tukey post hoc tests were used to evaluate significant main effects. In all cases, p < 0.05 was considered statistically significant. Data were analyzed with GraphPad Prism 5 software (GraphPad Software Inc, USA).

## Results

### Evaluation of the metabolic parameters of Swiss mice fed HFD

**Fig 1:**
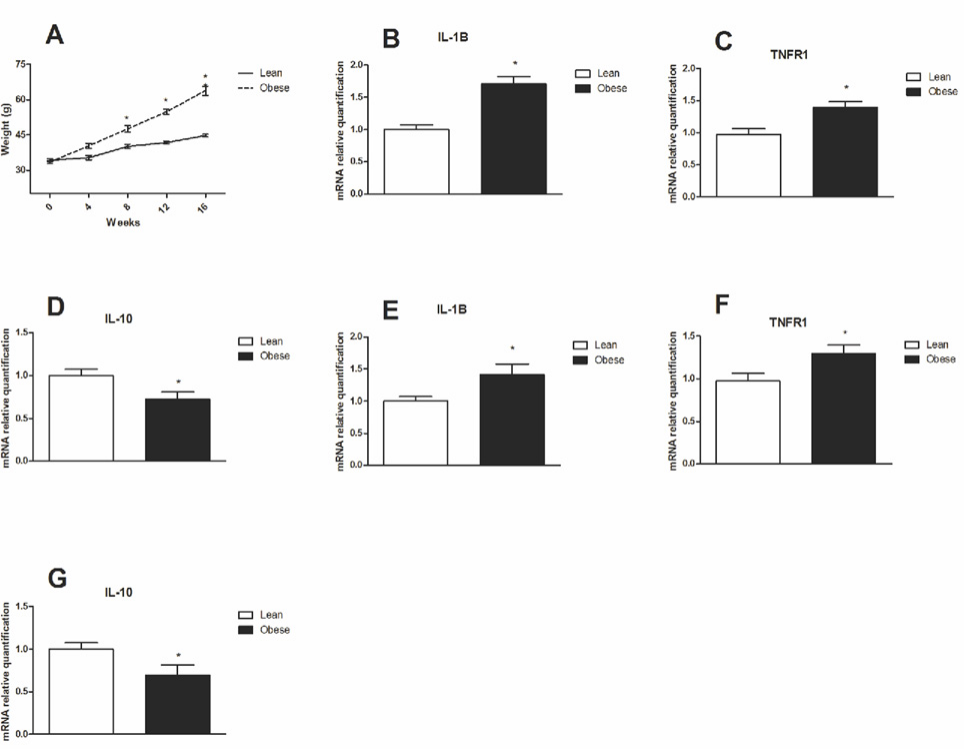
Weight gain and inflammatory markers in hypothalamus of obese mice. **A.** Weight gain of mice fed a high fat diet compared to chow for 16 weeks. QRT-PCR was used to analyze the gene expression of **B.** IL1-β, **C.** TNFR1 and **D.** IL-10 in the hypothalamus of DIO mice at 8 weeks of treatment. QRT-PCR was used to analyze the gene expression of **E.** IL1-β, **F.** TNFR1 and **G.** IL-10 in the hypothalamus of DIO mice at 16 weeks of treatment. Values are shown as mean ± SEM. *P < 0.05; n = 6 animals per group.

Swiss mice that were fed a HFD exhibited higher body mass than mice that were fed a control diet (Chow), from 8 to 16 weeks (Fig. 1A). The mice that were on a HFD for 8 and 16 weeks became obese and presented increased mRNA levels for inflammatory markers such as IL-1β (Fig. 1B and 1E, respectively) and TNFR1 (Fig. 1C and 1F, respectively) and decreased IL-10 expression (Fig. 1D) when compared with the control group.

### Autophagy marker LC3 is abundantly expressed in the arcuate nucleus

**Fig 2:**
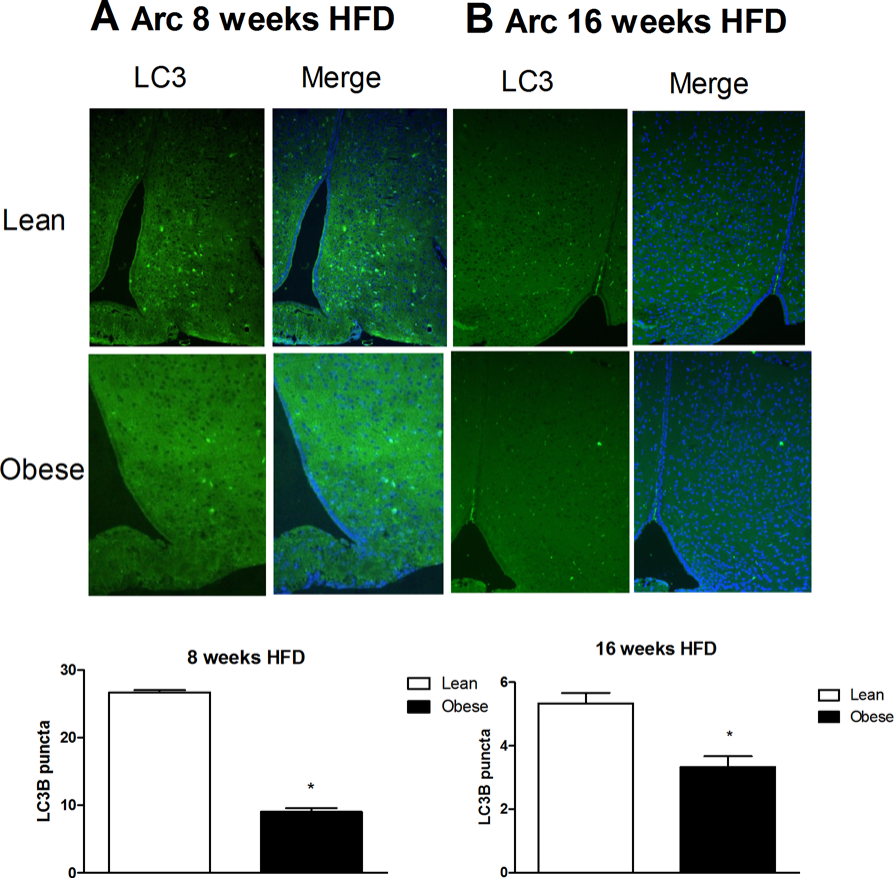
LC3 is expressed in the arcuate nucleus of Swiss mice and colocalizes with the hypothalamic neurons and microglia cells. **A.** Immunostaining of LC3 (green) in the arcuate hypothalamic nucleus of lean and obese mice (8 weeks of a HFD). **B**. Immunostaining of LC3 (green) in the arcuate hypothalamic nucleus of lean and obese mice (16 weeks of a HFD). DAPI was used for nuclear staining; n = 3 animals per group.

Autophagy is an important cellular process that degrades proteins and recycles organelles and pathogens. Alterations in autophagy flux can cause defects in insulin signaling and ER stress in peripheral tissues that contribute to obesity. In order to determine if autophagy regulation plays a role in hypothalamic cellular homeostasis, the presence of the autophagy marker LC3B (microtubule-associated protein light chain 3 B) was evaluated. As shown in Fig. 2, LC3B was expressed in the mediobasal hypothalamus of mice treated with chow and high fat diets for 8 (Fig. 2A) and 16 (Fig. 2B) weeks.

### Modulation of hypothalamic autophagy in mice chronically exposed to a saturated HFD

**Fig 3:**
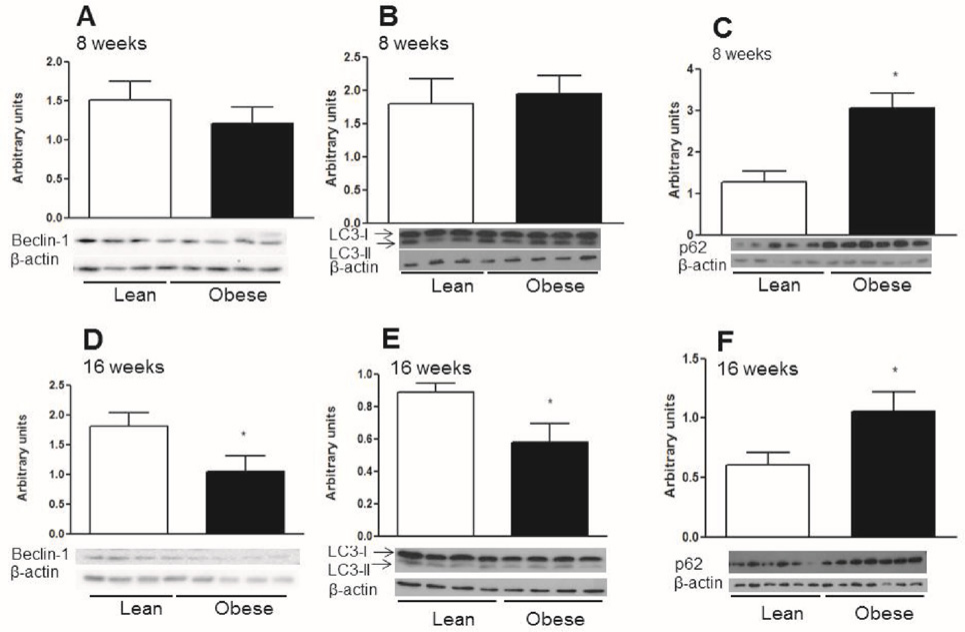
The contents of the autophagic proteins in the hypothalamus from diet induced obese mice with 8 and 16 weeks of treatment. **A.** Western-blotting shows the Beclin-1 to β-actin ratio in the hypothalamus of obese mice with 8 weeks on a HFD. **B.** Western-blotting shows LC3-II to β-actin ratio in the hypothalamus of obese mice with 8 weeks on a HFD. **C.** p62 content in the hypothalamus of obese mice with 8 weeks on a HFD. **D.** Beclin-1 content in the hypothalamus of obese mice with 16 weeks on a HFD. **E.** LC3-II to β-actin ratio in the hypothalamus of obese mice with 16 weeks on a HFD. **F.** p62 content in the hypothalamus of obese mice with 16 weeks on a HFD. Values are shown as mean ± SEM. *P < 0.05; n = 4–7 animals per group.

**Fig 4:**
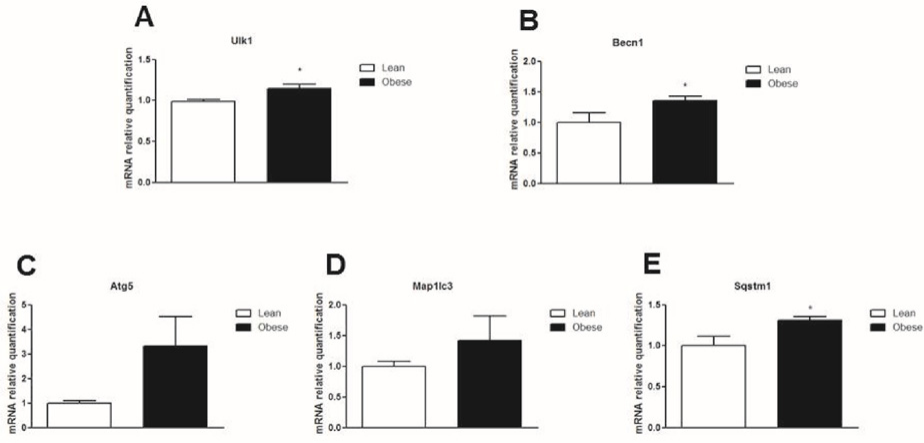
mRNA gene expression of autophagic proteins in the hypothalamus of obese mice. QRT-PCR was used to analyze the gene expression of **A.** Ulk1, **B.** BECN-1, **C.** ATG5, **D.** Map1lc3 and **E.** SQSTM1 in the hypothalamus of diet induced obese mice (16 weeks on a HFD) versus lean mice. Values are shown as mean ± SEM. *P < 0.05; n = 6 animals per group.

In order to determine if autophagy is regulated in obesity, Swiss mice were fed chow or a HFD for 16 weeks. After 8 weeks on a HFD, there were no differences in the protein content of Beclin-1 and LC3-II in the hypothalamus (Fig. 3A and 3B). However, at this 8 week time point, the protein content of p62 increased (Fig. 3C). This data shows that after 8 weeks on a HFD, autophagy was partially preserved in the hypothalamus. When mice were exposed to a 16 week HFD, the protein content of Beclin-1 and LC3-II decreased (Fig. 3D and 3E) while the protein content of p62 increased (Fig. 3F) when compared to lean mice. Gene expression of ULK1 BECN-1 and SQSTM1 were upregulated in obese animals (Fig. 4D, 4B and 4E, respectively). There were no differences in the LC3 gene, MAPLLC3, (Fig. 4D) when compared to the control chow group. These data demonstrated that impairment of the hypothalamic autophagy occurs when mice are chronically (16 weeks) unfed HFD. This leads to a loss of autophagy that potentially causes the accumulation of aggregated proteins and loss of cellular homeostasis.

### Rapamycin Increases hypothalamic autophagy and counteracts the HFD effects in mice

**Fig 5:**
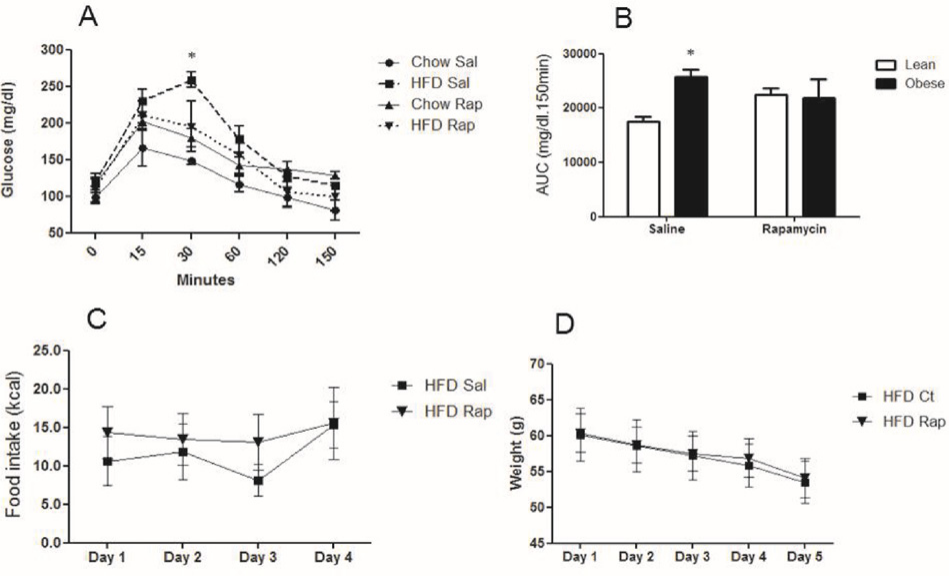
Metabolic parameters in mice treated with Rapamycin (icv). **A** and **B**. GTT curve and area under the GTT of lean or obese mice (16 weeks of a HFD) treated with 2 μl of a solution that contained 25 μM rapamycin (Rap) or saline (Sal) once a day for 6 days. **C and D.** Food intake and weight modulation in obese mice treated with rapamycin. HFD versus chow diet in mice treated with saline and a HFD in mice treated with rapamycin versus a HFD in mice treated with saline. Values are shown as mean ± SEM. *P < 0.05; n = 5–6 animals per group.

**Fig 6:**
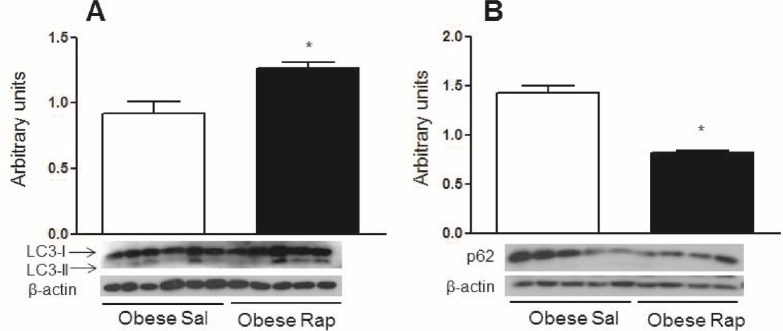
Rapamycin treatment reverses the autophagic downregulation in obese mice. **A.** Western blotting showing LC3-II to β-actin ratio in the hypothalamus of diet induced obese mice (16 weeks on a HFD) treated (once per day) for 5 days with 2 μl of a solution that contained 25 μM rapamycin (Rap) or saline (Sal). **B.** The p62 content in the hypothalamus of the diet induced obese mice treated with rapamycin. Values are shown as mean ± SEM. *P < 0.05; n = 5–6 animals per group.

**Fig 7:**
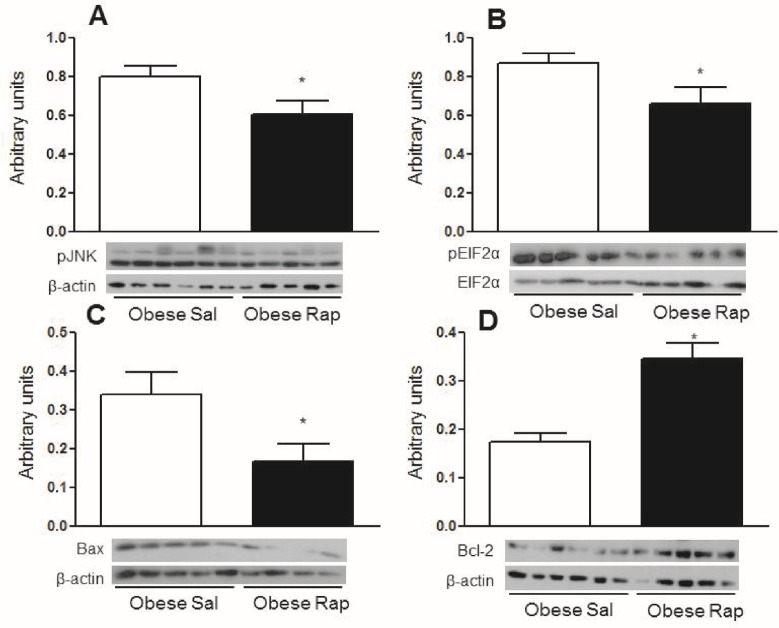
Rapamycin treatment decreases inflammation, endoplasmic reticulum stress and apoptosis markers in obese mice. **A and B.** Western blotting of pJNK and pEIF2α content in the hypothalamus of obese mice (16 weeks on a HFD) treated (once per day) for 5 days with 2 μl of a solution that contained 25 μM rapamycin (Rap) or saline (Sal). **C and D.** Bax and Bcl-2 content in hypothalamus of obese mice treated with rapamycin. Values are shown as mean ± SEM. *P < 0.05; n = 5–6 animals per group.

Since mice that were chronically fed a HFD showed defects in autophagy, obese mice were treated with rapamycin, an mTOR inhibitor that induces autophagy, to determine if activation of hypothalamic autophagy reverses the deleterious effects of obesity. Fig. 5A shows that 30 minutes after a glucose challenge, obese mice treated with a rapamycin icv injection had improved glucose levels. Obese mice treated with saline had higher glucose levels at this point than lean mice given the same saline treatment. However, no differences were observed in the total area under the curve for glucose in obese rapamycin-treated mice. The only observed differences were between the obese and lean saline-treated mice (Fig. 5B). There were no significant differences in food intake and body mass between the rapamycin-treated and untreated animals (Fig. 5C and 5D). While rapamycin treatment did not cause any significant differences in systemic glucose metabolism and food intake, this treatment increased the hypothalamic content of LC3-II (Fig. 6A) and decreased p62 (Fig. 6B). Rapamycin also reduced the phosphorylation of JNK1 and EIF2α (Fig. 7A and 7B, respectively), which are inflammatory and ER stress markers, respectively. Bax, a proapoptotic protein, (Fig. 7C) was reduced while Bcl-2, an antiapoptotic protein, (Fig. 7D) increased with rapamycin treatment; thus, the protective effects of autophagy in restoring cellular homeostasis were observed in the hypothalamus of the mice that were fed a HFD for 16 weeks.

### Saturated fatty acids modulate hypothalamic autophagy

**Fig 8:**
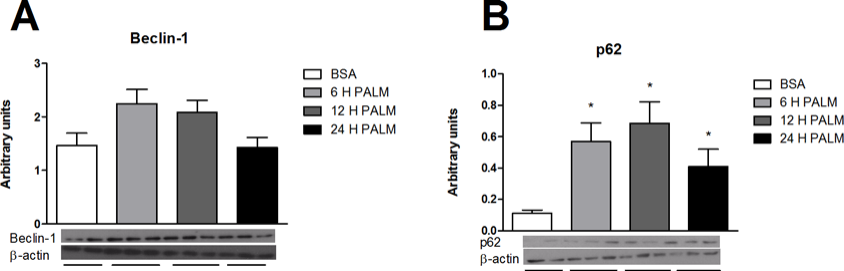
Effect of saturated fatty acid in a neuron cell line. **E**. Western-blotting shows the Beclin-1 and p62 to β-actin ratio, respectively in CLU-189 cell line treated with 500 μM of palmitic acid for 6, 12 or 24 hours. Values are shown as mean ± SEM. *P < 0.05;

**Fig 9:**
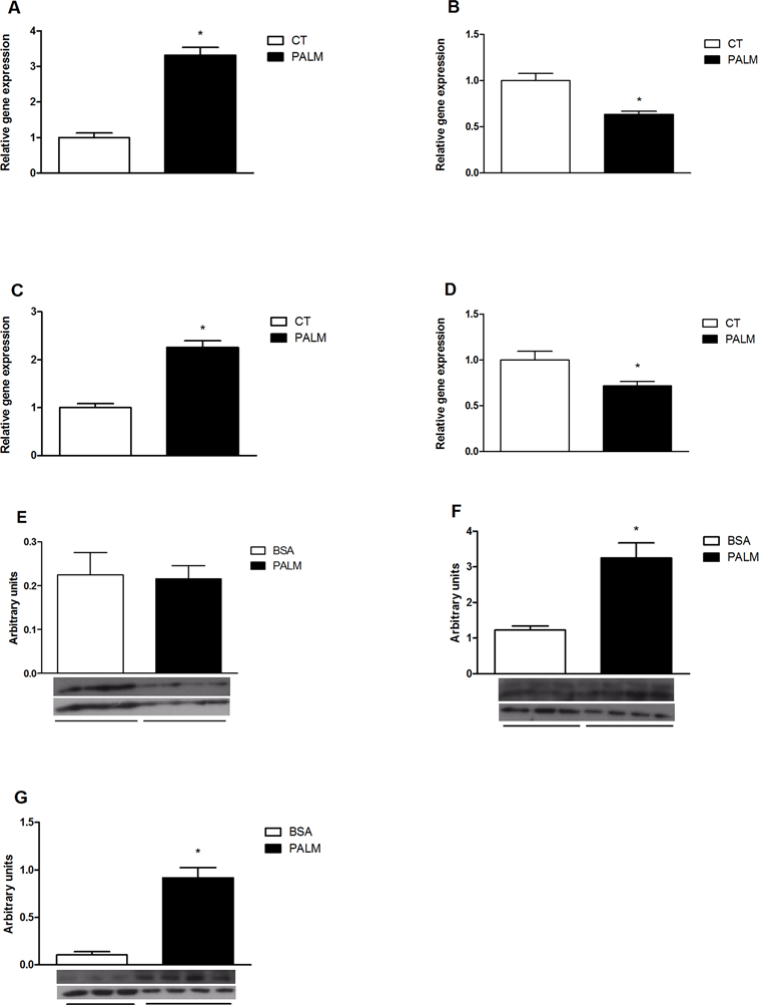
Effect of treatment with conditioned medium on hypothalamic neuron cell lien. **A, B, C and D.** QRT-PCR was used to analyze the gene expression of TNF, Map1lc3, Sqstm1, Becn1, respectively. **E**. Western-blotting shows the Beclin-1, LC3-II and p62 to β-actin ratio, respectively in CLU-189 cell line treated with conditioned medium of microglia cells BV-2 treated with 500 μM of palmitic acid for 6 hours. Values are shown as mean ± SEM. *P < 0.05;

Since long-chain saturated fatty acids have inflammatory properties in the hypothalamus [4], the effects of these fatty acids on autophagy were evaluated. We show that upon palmitate treatment a neuron cell-line (CLU-189), responds with an increase of p62 content (Fig. 8B). Then we tested the effect of a pre-conditioned medium collected from a microglial cell-line previously treated with palmitate on the regulation of inflammation and autophagy proteins in CLU-189. As shown in Fig. 9, there was an increased expression of TNF accompanied by a reduction of the expression of LC3B and beclin-1 and transient increase of p62 gene expression. Additionally, there was an increase in LC3B-II and p62 protein content (Fig. 9F and 9G).

## Discussion

Inflammation in peripheral tissues develops as a consequence of obesity; however, hypothalamic inflammatory signaling occurs in rats and mice 1 to 3 days after a HFD is consumed, prior to substantial weight gain. This indicates that diet rather than increased adiposity is the cause of insulin insensitivity and inflammation [17].

This study investigated the hypothesis that chronic exposure to a HFD could modulate autophagy in the hypothalamus of mice. Autophagy is the main mechanism that degrades and recycles damaged organelles and long-lived proteins and its role in the central nervous system (CNS) has been demonstrated in several neurodegenerative diseases [30–33].

Both lean and obese mice showed LC3B in cells of the hypothalamic arcuate nucleus. Hypothalamic modulation of autophagy, in response to obesity, could be nucleus or cell specific. Recent studies have shown that basal levels of autophagy are important in maintaining the functionality of the hypothalamic neurons. When autophagy is specifically inhibited in agouti-related protein (AgRP) neurons, the physiological increase in the expression of this neurotransmitter in response to fasting is abolished, which increases POMC and α-MSH and creates a lean phenotype in mice [34]. In contrast, ATG7 deletions in POMC neurons cause the formation of protein aggregates to occur because p62 accumulates in the neuron [35]. P62 is a multifunctional adapter protein that recruits ubiquitinated proteins to the autophagosome [36–37] and its increase is a sign that defective autophagy is occurring in the cell [38]. The lack of effective autophagy in POMC neurons also results in excessive food intake, which is caused partially by leptin resistance and decreased energy expenditure; thus, animals with defective autophagy in their POMC neurons, exhibit increased body mass [35]. Another study showed that accumulation of aggregated proteins marked with ubiquitin and p62, decreased axonal projections of POMC neurons and the same phenotype resulted when ATG7 was specifically deleted from POMC neurons [39].

The quantification of autophagy is difficult because this mechanism is a dynamic process that involves the formation and processing of protein biomarkers such as LC3-II. However, a reduction in LC3-II was observed in response to a 16 week exposure to a HFD but not to an 8 week exposure to a HFD. This data suggests that autophagy becomes less efficient with a more prolonged exposure to obesity. Previous studies have shown that the amount of time animals and humans are exposed to an obesogenic environment (i.e. the longer the time in which the individual remains obese, the more difficult it is to restore metabolic homeostasis [40, 41]. This important finding supports the theory that the loss of hypothalamic neurons involved in regulating energy homeostasis is one of the reasons that food intake is difficult to control in chronic obesity [14, 18].

Autophagy and apoptosis have many pathways in common and regulating both mechanisms is crucial in determining if a cell will or will not survive in response to cellular stress [42]. Autophagy usually protects the neurons from cell death in stressful situations [43–45], but autophagy can be considered a type of programmed cell death [46] that could contribute to the apoptosis of hypothalamic neurons when chronic exposure to a HFD occurs. In this context, obese mice with defects in hypothalamic autophagy can exacerbate IKKβ/NF-kB activation and potentiate inflammation present in DIO [21].

Genes involved in the initial steps of autophagy induction were upregulated in mice exposed to 16 weeks of a HFD. Posttranslational events could be involved in regulating autophagy in obesity or autophagy could be disrupted at a later point such as ATG5–12 conjugation, LC3 processing or autophagosome/lysosome fusion. Although the exact mechanism by which autophagy was downregulated in this model is unknown, another possibility is that inflammatory mediators produced primarily by microglia, led to decreased autophagy activation in neurons [47].

Although autophagy occurs at basal levels in all cells, diverse environmental stressors and nutrient deprivation are strong inducers of this degradative mechanism [48]. A key negative regulator of autophagy is the nutrient sensor mTOR [49, 50], which is inhibited by rapamycin and is the reason rapamycin is largely used as an autophagy inducer [51].

The results from this study show that short-time treatment with rapamycin ameliorated glucose tolerance in obese mice despite a lack of difference in body mass. We suspect that the effect of rapamycin on body mass depends on previous defect in the mTOR pathway, which can be found in the obese state. In addition, there is a previous study showing that in age-related adiposity the impact of rapamycin in body mass occurs after 2 weeks of icv treatment [52], which is a longer time than the one we employed in our studies. Rapamycin also modulated important autophagic markers in the hypothalamus, suggesting that an autophagy improved. A recent study suggested that treatment time with rapamycin is crucial to metabolic phenotype. In the initial weeks of intraperitoneal treatment, the data showed that mice were glucose intolerant, but at 20 weeks of treatment, rapamycin ameliorated their sensitivity to glucose [53]. The results from the previous study suggest that rapamycin injected directly into the hypothalamus has different effects on glucose sensitivity as compare to a systemic injection during a short treatment time.

As expected, in obese mice treated with icv rapamycin, the inflammatory and apoptotic stimuli were inhibited and the induction of ER stress was reversed, which demonstrates the protective effects of autophagy in mice fed a HFD. Autophagic induction contributes to limiting ER stress [54]. A previous study showed that suppression of hepatic gene ATG7 in mice results in increased ER stress and insulin resistance [55].

We demonstrated that neuron cell-line responds with increase of p62, which reflects a defect in autophagy regulation. Since neurons are known to be resistant to the pro-inflammatory actions of palmitate [56], we demonstrate that neuron cell-line treated with pre-conditioned medium collected from a microglial cell-line previously treated with palmitate show an increased expression of TNF accompanied by a reduction of the expression of LC3B and Beclin-1 and a transient increase of p62. Thus, it is clear that palmitate has a direct effect on autophagy in neurons, but lacks inflammatory activity, which is only achieved through the mediation of microglia.

Our study is the first to reveal that autophagic dysfunction in the hypothalamus may be due to the direct effects of saturated fatty acids. However, the cellular and molecular pathways underlying these effects have not been elucidated. Since sexual dimorphism was observed in LPS-induced inflammatory mediator production with males being more susceptible to bacterial sepsis [57], it would be interesting to study the modulation of autophagy also in females. The working hypothesis is that obesity causes downregulation of hypothalamic autophagy which contributes to neuronal dysfunction and leads to the formation of protein aggregates, accumulation of dysfunctional mitochondria, inflammation and ultimately apoptosis. Perturbation of autophagic homeostasis could be related to apoptosis, which is present in the hypothalamic neurons of obese mice and is considered the irreversible cause for the loss of hypothalamic feed control in chronic obesity. Reversal of basal autophagy in the hypothalamus of obese mice is a potential target to control neuronal injury and improve glucose homeostasis. Understanding the biological basis of obesity and obesity-related pathologies and discovering medical therapies to restore metabolic function is essential for the biomedical community.
